# Integrative analysis of non-small cell lung cancer identifies Jumonji domain-containing 6/ETS homologous factor axis as a target to overcome radioresistance

**DOI:** 10.1038/s41392-025-02471-w

**Published:** 2025-12-01

**Authors:** Manni Wang, Li Xu, Aqu Alu, Peiheng Li, Jian Liu, Siyuan Chen, Xuemei He, Xuejiao Han, Li Yang, Qiang Pu, Xiawei Wei

**Affiliations:** 1 Laboratory of Aging Research and Cancer Drug Target, State Key Laboratory of Biotherapy and Cancer Center, National Clinical Research Center for Geriatrics, West China Hospital, Sichuan University, Chengdu, China; 2 Department of Biotherapy, Cancer Center, West China Hospital, Sichuan University, Chengdu, China; 3 Division of Thyroid Surgery, Department of General Surgery, West China Hospital, Sichuan University, Chengdu, China; 4 Department of Thoracic Surgery, Sichuan University West China Medical Center, Chengdu, China

**Keywords:** Cancer therapy, Cancer stem cells

## Abstract

Radiation therapy (RT) is a key treatment strategy for lung cancer, yet its efficacy is frequently compromised by radioresistance. The combination of RT with targeted therapies enhances treatment outcomes for non-small cell lung cancer (NSCLC). This study aims to investigate new mechanisms of metastasis after RT for NSCLC and improve the durability of the benefits of radiotherapy for lung cancer patients. This integrative study utilized human NSCLC tissue arrays, bulk RNA-sequencing, CUT&Tag sequencing, and single-cell RNA-sequencing to identify gene alterations induced by RT. In vitro experiments and animal studies were used to investigate the role of Jumonji domain-containing 6 (JMJD6)/ETS homologous factor (EHF) axis in post-RT metastasis of NSCLC. RT triggered the upregulation of JMJD6 in NSCLC tissues. This upregulation led to the activation of EHF and the subsequent transcription of pluripotency factor genes through the demethylation of H4R3me2s. JMJD6/EHF axis plays a critical role in NSCLC cell metastasis, potentially through the TGF-β/SMAD and AKT/ERK signaling pathways. These findings suggest JMJD6 as a potential therapeutic target to combat post-RT metastasis in NSCLC.

## Introduction

According to the latest GLOBOCAN reports, lung cancer remains the predominant cause of cancer-related mortality globally, which ranks as the second most prevalent malignancy across both genders, with a standardized cumulative lifetime risk estimated at 3.8% for males and 1.77% for females.^1,2^ Radiation therapy (RT) is a proven modality for local tumor control across various stages of lung cancer, with approximately 77% of patients having evidence-based indications for RT treatment. ^3^ The extensive use of RT has been associated with an 8.3% increase in 5-year local control rates and a 4% improvement in prognosis.^4^ However, the long-term benefits of RT are often compromised by radioresistance, a frequent clinical observation that results in tumor recurrence. The development of targeted therapies has brought significant improvement in the prognosis of lung cancer over the past decades, particularly for those afflicted with non-small cell lung cancer (NSCLC). A recent analysis indicates that the incorporation of targeted therapies has contributed to a reduction in the mortality rate of NSCLC patients.^5^ Despite these advances, the challenge of intrinsic and acquired resistance to targeted agents persists, and the complex interplay between these target therapies and radiotherapy is an area of intense investigation. It is thus imperative to dissect the molecular mechanisms underpinning the radioresistance of lung cancer, and to explore the therapeutic efficacy of integrating targeted therapies with RT.

Emerging evidence over recent years has implicated a specific subpopulation within the heterogeneous tumor mass, characterized by self-renewal and multilineage differentiation capabilities, as a key contributor to treatment failure. This subpopulation, known as cancer stem-like cells (CSCs), has been extensively documented.^6–8^ CSCs are responsible for tumor initiation, progression, and metastasis. More critically, they possess enhanced capabilities for DNA damage repair, activated pro-survival signaling pathways, and a relative dormancy that protects them from conventional therapies that typically target rapidly dividing cells. Recent studies have revealed that radiation exposure can induce the trans-differentiation of non-stem cancer cells into CSCs, thereby endowing the tumor with enhanced radioresistance.^9,10^ The enrichment of CSCs following radiotherapy creates a vicious cycle: treatment designed to kill cancer cells may inadvertently expand the most treatment-refractory cellular compartment, leading to tumor repopulation and relapse. The abundance of CSCs during the course of treatment has been established as a significant prognostic indicator and a pivotal factor influencing the efficacy of fractionated RT in achieving local tumor control.^11,12^ Therefore, therapeutic strategies capable of eradicating CSCs or preventing their induction hold immense promise for breaking this cycle and achieving lasting therapeutic success. Despite research progress achieved, the underlying mechanisms of CSC-mediated radioresistance are not yet fully elucidated. Elevated expressions of stemness-associated genes, including CD44, CD133, OCT4, and ALDH1, are correlated with radioresistance and adverse patient outcomes.^13,14^ These markers are not merely identifiers but are functionally involved in maintaining stemness, regulating symmetric and asymmetric division, and interacting with niche microenvironments that promote survival. However, the upstream regulatory networks that control the expression and function of these stemness genes remain a central and incompletely answered question in the field.

In addition to gene mutations, epigenetic modifications such as histone methylation, acetylation, and ubiquitination also exert pivotal influences on the regulation of stemness genes.^15^ Unlike genetic alterations, epigenetic changes are dynamic and reversible, making them attractive therapeutic targets. They allow cancer cells to rapidly adapt to environmental stresses, such as radiation, by altering the chromatin landscape and transcriptional programs without changing the underlying DNA sequence. A study characterized JMJD6 as an arginine demethylase specific for histone H3 at arginine 2 (H3R2) and histone H4 at arginine 3 (H4R3).^16^ By removing methyl groups from these histone residues, JMJD6 can alter chromatin structure and gene accessibility, thereby regulating the epigenetic transcription of target genes. The aberrant expression of JMJD6 is involved in the etiology and progression of a spectrum of malignancies, such as melanoma,^17^ prostate^18^ and breast cancer.^19^ In the context of lung cancer, JMJD6 is postulated to act as an oncogene, facilitating epithelial-mesenchymal transition (EMT), a process closely linked to the acquisition of stem-like properties, yet its specific contribution to the modulation of tumor response to radiation therapy warrants further investigation.^20^

This study revealed an upregulation of JMJD6 in radioresistant non-small cell lung cancer (NSCLC) cells and in patient tumors, which correlates with a diminished prognosis and an elevated risk of metastasis. We hypothesize that JMJD6 drives radioresistance in NSCLC by epigenetically reprogramming cancer cells towards a stem-like state. Through human NSCLC tissue arrays, CUT&Tag sequencing, bulk RNA-sequencing, single-cell RNA-sequencing, and a series of in vitro and in vivo experiments, we aim to identify the key downstream transcriptional targets, such as EHF, through which JMJD6 exerts its effects, and to evaluate the radiosensitizing potential of genetic inhibition of JMJD6, providing a rationale for a novel combinatorial therapeutic strategy to overcome treatment failure in post-RT NSCLC patients.

## Results

### Upregulated JMJD6 expression correlates with poor prognosis and tumor metastasis in NSCLC patients

**Fig. 1:**
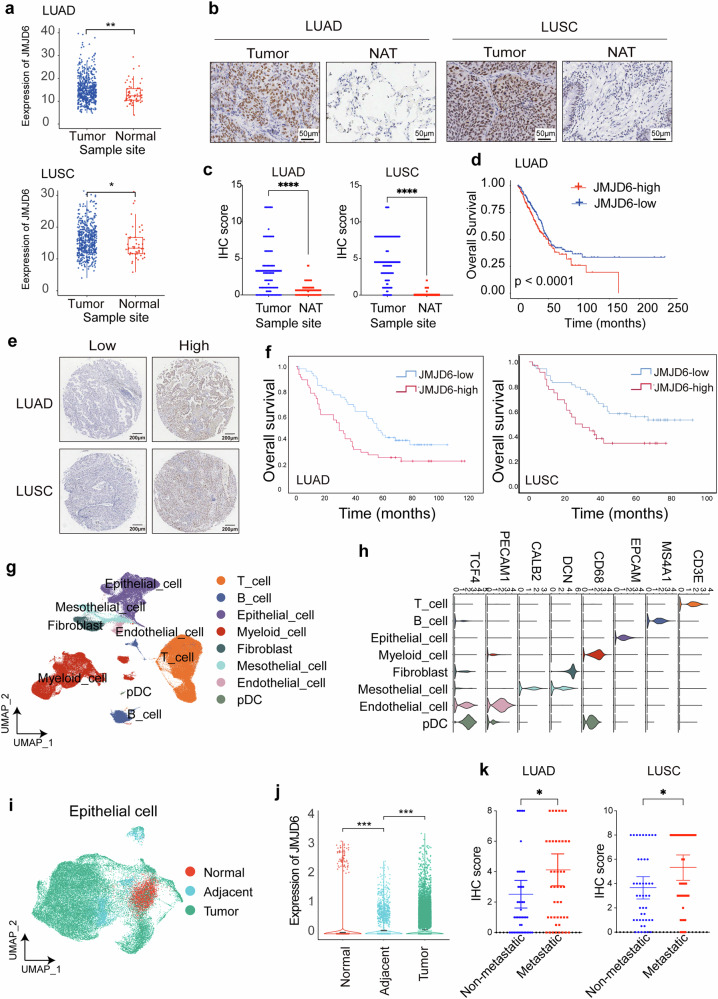
JMJD6 expression level is upregulated in NSCLC tissues and positively associated with OS of NSCLC patients. **a** The boxplots displaying JMJD6 expression in tumor and normal tissues of lung adenocarcinoma (LUAD) and lung squamous cell carcinoma (LUSC) based on TCGA data. **b** Higher JMJD6 expression levels were detected in tumor tissue samples compared with their paired adjacent noncancerous tissues (scale bar = 50 μm) in our patient cohort (*n* = 172), with statistical significance detected with **c** Wilcoxon matched-pairs signed-rank test. Data shown as mean ± SD, ****p < 0.0001. **d** The survival curve of the JMJD6 in LUAD patients based on the RNA-seq data and survival information retrieved from TCGA database, *p* < 0.0001. **e** JMJD6 expression of tumor tissues was characterized as high and low according to expression score (scale bar = 200 μm). **f** Kaplan–Meier analysis of our patient cohort (n = 188) suggested that patients with high JMJD6 levels had shorter overall survival (OS) compared with those with low JMJD6 levels. **g** scRNA-seq of publicly available datasets was integrated. Uniform manifold approximation and projection (UMAP) plots of a total of 202449 cells, characterized into 8 main cell populations (T cells, B cells, epithelial cells, myeloid cells, fibroblasts, mesothelial cells, endothelial cells, and pDC). **h** Violin plots showing the expression levels of the cellular components-specific markers. **i** UMAP plots of epithelial cells derived from normal, adjacent, tumor, and PE samples. **j** JMJD6 expression in epithelial cells of normal, adjacent, and tumor samples. **k** NSCLC patients with metastasis displayed a higher tumor JMJD6 expression compared with those of non-metastatic patients. Data shown as mean ± SD, *p < 0.05. NAT: histologically normal tissue adjacent to the tumor

The boxplots display JMJD6 expression in tumor and normal tissues from other cancer types based on RNA-sequencing data derived from TCGA. To identify the role of JMJD6 in lung cancer, we retrieved RNA-seq data of lung adenocarcinoma (LUAD) and lung squamous cell carcinoma (LUSC) patients from TCGA. The expressions of JMJD6 were elevated in both tumor samples (*p* = 0.002 for LUAD, *p* = 0.045 for LUSC) (Fig. 1a). As the aberrant expression of JMJD6 is closely related to the occurrence and development of various cancers, including melanoma,^17^ prostate^18^ and breast cancer,^19^ we have also analyzed JMJD6 expression of other cancer types (Supplementary Fig. 1). To confirm the results analyzed based on TCGA data, we further conducted IHC analyses in a NSCLC patients cohort comprised of 188 NSCLC patients (90 LUSC patients and 98 LUAD patients) to evaluate the clinical significance of JMJD6 in NSCLC. Among the 188 NSCLC patients included in the survival analysis, 172 patients were enrolled with paired tumor tissues and adjacent normal tissues for the comparison of JMJD6 expression. As detected by IHC, JMJD6 expression was significantly upregulated in tumor tissues compared with their paired adjacent noncancerous tissues (NAT) (Fig. 1b). The Wilcoxon matched-pair signed rank test results further confirmed the significant upregulation of JMJD6 in tumor tissues compared with their noncancerous counterparts (Fig. 1c). Furthermore, based on the RNA-seq data and survival information retrieved form TCGA database, LUAD patients with higher expression of JMJD6 demonstrated decreased survival rates (*p* < 0.001) (Fig. 1d). Survival curves of patients with other cancer types using TCGA dataset was shown in Supplementary Fig. 2. We then confirmed the correlation between tumor expression levels of JMJD6 with prognosis in our patient cohort. The median JMJD6 expression scores were used as the cut-off value for JMJD6 expression, and the example of high and low expression was shown in Fig. 1e and Supplementary Fig. 3. Based on the follow-up results between May 2008 and July 2018, the overall survival (OS) time of LUSC patients with low and high JMJD6 expression was 51.0 ± 25.4 and 28.0 ± 23.0 months respectively, whereas in LUAD, patients with low and high JMJD6 expression had OS of 56.5 ± 27.3 and 30.0 ± 31.9 months respectively. Results from Kaplan–Meier survival analysis suggested that NSCLC patients with higher JMJD6 expression had worse prognosis compared to those with low tumor expression of JMJD6 (Fig. 1f). Elevated JMJD6 expression levels were significantly associated with shorter OS in LUSC (log-rank test, χ2 = 5.783, *p* = 0.016) and LUAD patients (log-rank test, χ2 = 5.915, *p* = 0.015).

To characterize the epithelial cells of the tumor microenvironment (TME), we acquired and integrated the publicly released scRNA-seq profiles of lung tumors.^21–25^ A scRNA-seq atlas was built from 18 tumors samples, 15 pleural effusion samples, 11 adjacent samples, and 2 normal samples collected from 33 individuals. Following quality filtering and batch-effect removal, more than 200000 cells were included for analysis (Fig. 1g). Eight main cell populations were identified through canonical markers, including T cells (CD3E), B cells (MS4A1), epithelial cells (EPCAM), myeloid cells (CD68), fibroblasts (DCN), mesothelial cells (CALB2), endothelial cells (PECAM1), and pDC (TCF4) (Fig. 1h). Epithelial cells were extracted and subjected to secondary clustering. The distributions of samples of epithelial cells were illustrated in Fig. 1i. Notably, increased JMJD6 expression was observed in tumor samples in contrast to epithelial cells in the adjacent and normal samples (Fig. 1j). In addition, we evaluated whether JMJD6 expression was associated with tumor metastasis status. Results suggested higher tumor expression of JMJD6 in patients with tumor metastasis compared with that of non-metastatic patients (*p* = 0.017 in LUAD and *p* = 0.012 in LUSC), indicating that tumor JMJD6 expression is positively associated with metastasis incidence of NSCLC (Fig. 1k).

### Radiotherapy upregulates JMJD6 expression in NSCLC

**Fig. 2:**
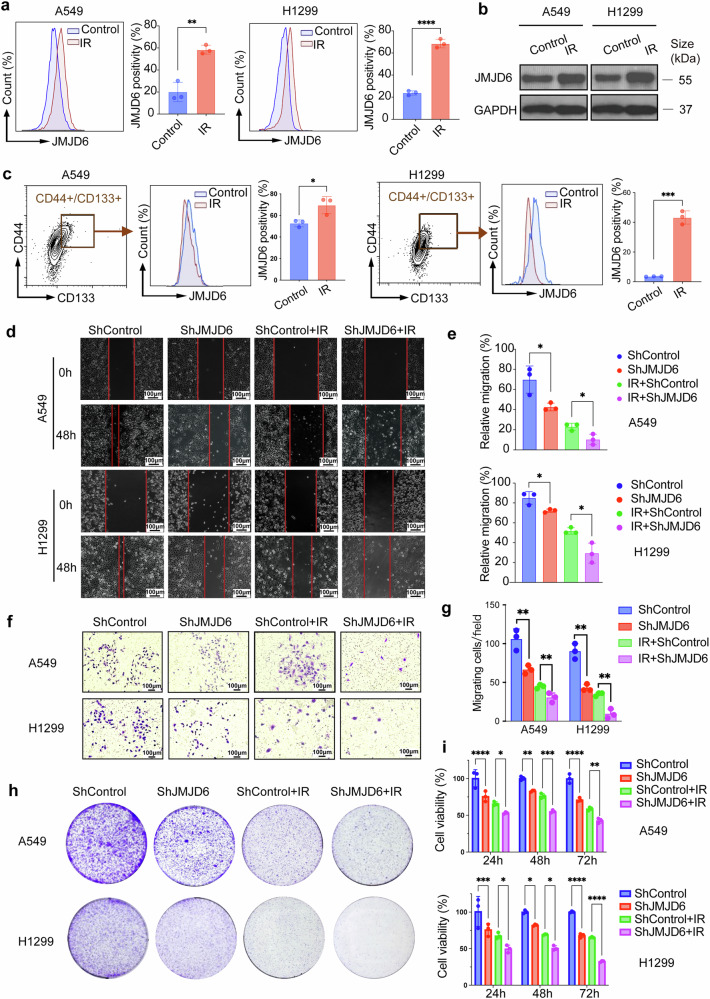
JMJD6 is upregulated by IR and promotes post-RT metastasis and proliferation of NSCLC in vitro. **a** Flow cytometry assays demonstrating the increased expression of JMJD6 in A549 and H1299 cells receiving IR. Data shown as mean ± SD, **p < 0.01, ****p < 0.0001. **b** Single representative Western blot assay showing JMJD6 protein levels in A549 and H1299 cells receiving IR. **c** Flow cytometry assays showing elevated expression of JMJD6 in CSC subpopulations (CD44 + CD133 + ) of NSCLC cells. Data shown as mean ± SD (standard deviation), *p < 0.05, ****p < 0.0001. **d** Representative images of wound healing assays on A549 and H1299 cells with JMJD6 knockdown, and images were taken at 0 and 48 h after wounds (scale bar = 100 μm). **e** Quantification analyses of relative wound migration. Data shown as mean ± SD, *p < 0.05. **f** Representative images of transwell assays showing cell migration of A549 and H1299 cells with JMJD6 knockdown (scale bar = 100 μm). **g** The quantification analyses of migrated cells/field. Data shown as mean ± SD, **p < 0.01. **h** Representative images of colony formation JMJD6 knockdown decreased the colony formation formed by A549 and H1299 cells receiving RT. **i** CCK-8 cytotoxicity tests assessing the cell viability of JMJD6-knockdown A549 and H1299 cells at 24, 48, and 72 h post-RT. Data shown as mean ± SD, *p < 0.05, **p < 0.01, ***p < 0.001, and ****p < 0.0001

To explore the effect of radiotherapy on JMJD6 expression, we treated NSCLC cell lines A549 and H1299 with ionizing radiation (IR), followed by 24 h incubation. To identify the optimal dosage of human NSCLC cells, we performed multi-dose clonogenic assays (0/2/5/8 Gy) in A549 cells, indicating that 5Gy of radiation is able to induce considerable anti-tumor effect (Supplementary Fig. 4). Given the localization of JMJD6 in both cytoplasm and the nucleus, the global expression of JMJD6 in NSCLC cells was examined by flow cytometry (Fig. 2a) and Western blot assays (Fig. 2b), which suggested elevated JMJD6 expression in both cell lines. IR induced an increase in the total JMJD6-positivity in both A549 cells and H1299 cells. At the same time, a subgroup of cells that highly express CD44 and CD133, two common surface markers for lung cancer stem cells, was selected with flow cytometry assays and detected for JMJD6 positivity. Compared with the untreated cells, the JMJD6-positive fractions were significantly elevated in CSC subpopulations (CD44 + CD133 + ) induced by IR (Fig. 2c).

### JMJD6 knockdown inhibits post-RT metastasis and proliferation of NSCLC in vitro

To explore the effect of JMJD6 on NSCLC metastasis, we first used JMJD6-targeting shRNA to induce specific knockdown of JMJD6 in H1299 and A549 cell lines. The knockdown efficiency of JMJD6-targeting shRNA in H1299 and A549 cells was detected with Western blot assays. Cell mobility represents the initial step of tumor metastasis. The wound healing and transwell assays were performed to determine the role of JMJD6 in NSCLC cell metastasis. Compared with untreated control cells, IR reduced the wound gap closure rates of both A549 and H1299 cells. In groups treated with IR, the mean relative wound healing was further reduced in the control group in JMJD6-knockdown group in H1299 cells and A549 cells (Fig. 2d, e). Likewise, transwell assays also demonstrated reduced migratory ability of A549 and H1299 cells by JMJD6 knockdown (Fig. 2f, g). To verify that JMJD6 depletion inhibits murine cell motility, we conducted transwell assays using mouse lung cancer cell lines LL/2 and CMT-64 (Supplementary Fig. 5). Clonogenic assays further revealed that JMJD6 depletion radiosensitized NSCLC cells, as evidenced by decreased colony formation in shJMJD6 A549 and shJMJD6 H1299 cultures than in shControl counterparts (Fig. 2h), an equivalent anti-proliferative effect was observed in LL/2 and CMT-64 cells (Supplementary Fig. 6). CCK-8 cytotoxicity tests were performed on A549-shJMJD6 and H1299-shJMJD6 cells treated with RT. Although RT markedly lowered cell survival at 24, 48, and 72 h after exposure, ShJMJD6 knockdown further amplified radiation-induced suppression of proliferation (Fig. 2i). Overall, these results suggested that JMJD6 could promote metastasis, and knockdown of JMJD6 could further reduce NSCLC cell migration after RT.

### JMJD6 promotes post-RT enrichment of CSC subpopulations of NSCLC

**Fig. 3:**
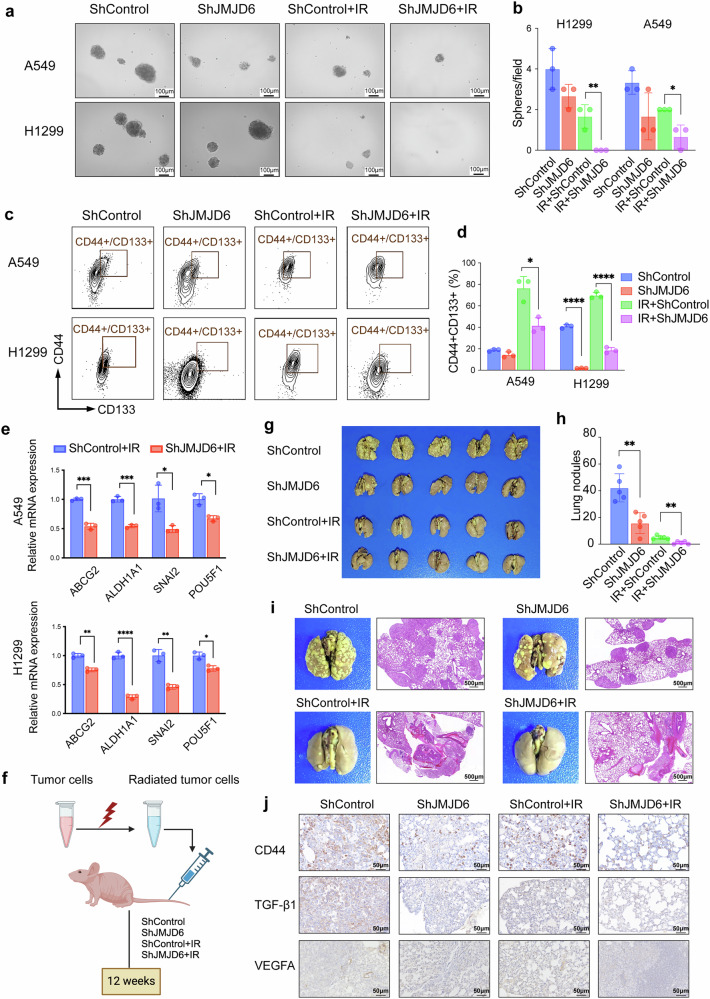
JMJD6 knockdown inhibits post-RT enrichment of CSC subpopulations and metastasis of NSCLC in vivo. JMJD6 promotes post-RT enrichment of CSC subpopulations of NSCLC (**a**–**e**). **a** The sphere formation assay of A549 and H1299 cells showing reduction of post-RT sphere-forming capacity by JMJD6 knockdown (scale bar = 100 μm). **b** The quantification analyses of tumor spheres larger than 50μm/field. Data shown as mean ± SD, *p < 0.05, **p < 0.01. **c** The CD44 + CD133+ dual positive fractions of shControl or shJMJD6 A549 and H1299 cells detected by flow cytometry. **d** Quantification analyses of CD44 + CD133+ subpopulations. Data shown as mean ± SD, *p < 0.05, ****p < 0.0001. **e** The qPCR assays assessing mRNA levels of stemness-related markers in A549 and H1299 cells. Data shown as mean ± SD, *p < 0.05, **p < 0.01, ***p < 0.001, ****p < 0.0001. **f** IR-treated shControl or shJMJD6 A549 cells were injected through the lateral tail vein of BALB/C nude mice. Mice were sacrificed on the 12th week after injection, and lung metastases were evaluated by gross morphology of the lungs and the number of metastatic nodules on the lung surface. **g** Formation of metastatic nodules in the lungs of mice on the 12th week after A549 cell injection (*n* = 5 mice per group). **h** The quantification analyses of metastatic lung nodules. Data shown as mean ± SD, **p < 0.01. **i** Representative images of metastatic nodules and the H&E staining for JMJD6 in lungs with A549 tumors (scale bar = 500 μm). **j** Representative IHC staining of CD44, TGF-β1, and VEGFA1 in mouse lungs with shControl or shEHF A549 tumors (scale bar = 50 μm)

As JMJD6 was upregulated in CSC-enriched populations, we investigated whether the knockdown of JMJD6 could inhibit the stemness properties of lung CSCs and the expression of pluripotency factor genes. Lung CSCs are characterized by high CD133 and CD44 expression. The IR-treated shJMJD6 NSCLC cells derived a decreased number of tumor spheres compared to shControl cells (Fig. 3a, b). We then conducted flow cytometry assays to evaluate the CD44 CD133 double-positive fraction of IR-treated NSCLC cells. IR induced a 2–4-fold increase in the CD44CD133 double-positive fractions of NSCLC cells, whereas JMJD6 knockdown suppressed this increase (Fig. 3c, d). The mRNA level of stemness-related genes was then assessed through qPCR to evaluate JMJD6-mediated CSC characteristics in NSCLC cells. As shown in Fig. 3e, both A549-shJMJD6 cells and H1299-shJMJD6 cells expressed lower mRNA levels of stemness-related markers, including ABCG2, ALDH1A1, POU5F,1 and SNAI2 than their shControl counterparts. The collective results indicated that JMJD6 functions as a positive regulator of stem-like properties in NSCLC cells post-radiotherapy.

### JMJD6 knockdown inhibits post-RT metastasis of NSCLC in vivo

An experimental lung metastatic mouse model was established to investigate whether JMJD6 promotes the metastasis of NSCLC cells in vivo. IR-treated shControl or shJMJD6 A549 cells were injected through the lateral tail vein of BALB/C nude mice, and mice were sacrificed on the 12th week after injection (Fig. 3f). Lung metastases were evaluated by gross morphology of the lungs and the number of metastatic nodules on the lung surface (Fig. 3g). Mice injected with shControl A549 cells developed increased numbers of lung nodules compared with those injected with shJMJD6 A549 cells (Fig. 3g, h). The same suppressive effect of JMJD6 depletion was seen when A549 cells were pre-treated with irradiation (Fig. 3h), indicating that JMJD6 knockdown enhances the efficacy of radiotherapy. A similar trend was obtained in a syngeneic model: mice inoculated with shJMJD6 LL/2 murine lung cancer cells likewise exhibited markedly fewer pulmonary metastatic foci than the shControl group (Supplementary Fig. 7).

The microscopic lung metastatic nodules were assessed with hematoxylin-eosin (H&E) staining of lung tissues. Knockdown of JMJD6 in A549 cells reduced the metastasis incidence of lung cancer regardless of IR treatment (Fig. 3i). Immunohistochemistry staining for CD44, TGF-β, and VEGFA1 was performed in lung tissues of A549 tumor-bearing mice, which revealed that regardless of RT, JMJD6-knockdown tumors had decreased positivity of CD44, TGF-β1 and VEGFA1 compared with control tumor (Fig. 3j). These results suggested JMJD6 expression in NSCLC tumors is positively associated with cancer stemness, angiogenesis and metastatic abilities of tumors.

### JMJD6 promotes EHF transcription through the demethylation of H4R3me2s at the EHF promoter

**Fig. 4:**
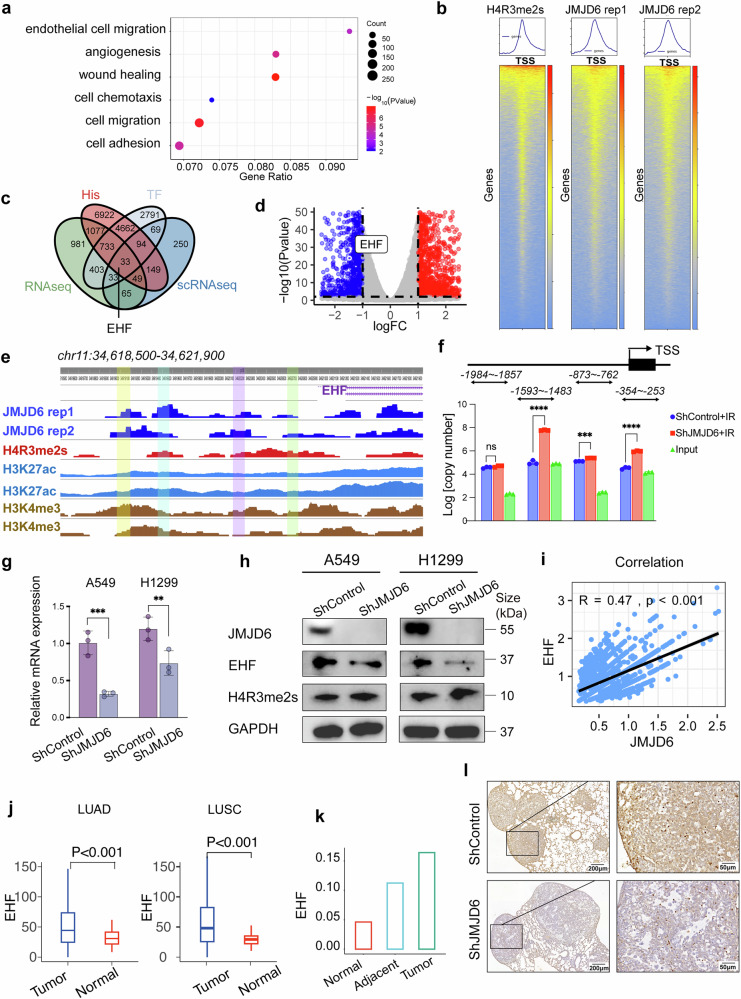
JMJD6 binds to EHF promoter and regulates EHF transcription through the demethylation of H4R3me2s. **a** The GO pathway enrichment analysis of the metastasis-related DEGs based on RNA-seq results in A549 cells transfected with shControl and shJMJD6. **b** CUT&Tag analyses measuring the levels of JMJD6 and H4R3me2s bound at the TSS in A549 cells. **c** The Venn diagram showing the overlapped the RNA-seq data, CUT&Tag data, and highly expressed genes in tumor samples calculated from scRNA-seq data. **d** The volcano plot of EHF, which was selected as a downstream target of JMJD6 among the overlapping target genes. **e** The IGV plots showing JMJD6, H4R3me2s, H3K4me3 and H3K27ac binding density on EHF in A549 cells. Data obtained from Encodeproject (www.encodeproject.org). The significant variation regions are labeled. **f** CUT&Tag-qPCR assays identifying H4R3me2a binding region at the EHF promoter in JMJD6-knockdown or JMJD6-intact A549 cells. Primers used for the qPCR assays are shown in the supplementary files. RT-qPCR (**g**) and Western blot (**h**) showing the reduced expression level of EHF and a global increase in H4R3me2s expression of JMJD6-knockdown A549 cells. Data shown as mean ± SD, *p < 0.05, **p < 0.01. **i** Integrated scRNA-seq dataset showing the correlation between JMJD6 and EHF expressions in epithelial cells. **j** The boxplots displaying EHF expression of tumor and normal samples of LUAD and LUSC derived from TCGA dataset. **k** Integrated scRNA-seq dataset showing the expression of EHF in epithelial cells of normal, adjacent, and tumor samples. **l** Representative images of IHC staining for EHF in mouse lungs with shJMJD6 and shControl A549 tumors, scale bar = 200 μm (left panel) and 50 μm (right panel)

To investigate the underlying mechanism through which JMJD6 promotes NSCLC metastasis, we conducted transcriptome profiling analyses of A549 cells transfected with shControl and shJMJD6. The sequencing analysis revealed 2399 differentially expressed genes (DEGs) between the two groups (log2FoldChange > 1 or <0.01), of which 1475 genes were upregulated and 924 genes were downregulated upon JMJD6 knockdown. The GO pathway enrichment analysis suggested that the differentially expressed genes were significantly enriched in pathways related to tumor metastasis such as angiogenesis, cell motility, cell migration, and stem cell maintenance (Fig. 4a). To identify genes regulated by JMJD6, we next performed CUT&Tag sequencing of JMJD6 in IR-treated A549 NSCLC cells (Fig. 4b). As JMJD6 has long been identified as a demethylase for histone H4 at arginine 3 (H4R3), and H4R3me2 is a key epigenetic event in cancer,^16,26^ the binding of symmetric H4R3me2 on A549 genome was also assessed. By intersecting the DEGs identified from RNA-seq and CUT&Tag sequencing, and highly expressed genes in tumor samples calculated from scRNA-seq data, the overlapping targets were selected for qPCR validation (Fig. 4c). Among the overlapping target genes, EHF, the expression of which is correlated with increased metastasis of lung cancer cells is by far one of the most downregulated overlapping targets of JMJD6 (Fig. 4d). The CUT&Tag sequencing data demonstrated the binding distributions of JMJD6 on EHF promoter (Fig. 4e), which showed that JMJD6 directly bound on the transcription start site (TSS) regions of EHF locus. As H3K4me3 and H3K27ac are chromatin modifications at the transcription start site of active genes in eukaryotes and are known as promoter chromatin tags,^27–29^, we also assessed the H3K4me3 and H3K27ac peaks near TSS regions of EHF in A549 cells using data from Encodeproject (www.encodeproject.org). The binding sites of H4R3me2s, H3K27a,c and H3K4me3 sites were overlapped with those of JMJD6 binding (Fig. 4e). Given that the histone H4 at arginine 3 is a preferred substrates for JMJD6 demethylase activity, and that H4R3me2s modification is associated with transcription repression,^30^ we then investigated whether the demethylation of H4R3me2 on the TSS region of EHF was regulated by JMJD6. Consistent with CUT&Tag results, the quantitative PCR revealed increased enrichment of H4R3me2s at the EHF promoter in JMJD6-knockdown A549 cells compared with the JMJD6-intact counterpart (Fig. 4f). RT-qPCR analyses (Fig. 4g) and Western blot (Fig. 4h) also suggested a reduced level of EHF in JMJD6-knockdown A549 cells, further supporting the regulatory role of JMJD6 in EHF expression. Western blot assays showed that JMJD6 depletion led to a global increase in H4R3me2s expression, indicating that JMJD6 is a key demethylase for H4R3me2s in A549 cells.

Results form scRNA-seq also demonstrated a positive correlation between JMJD6 and EHF expression levels in epithelial cells (R = 0.47, *p* < 0.001) (Fig. 4i). The TCGA-derived RNA-seq profiles proved that EHF was highly expressed in tumor samples of LUAD and LUSC patients (*P* < 0.001, *P* < 0.001) (Fig. 4j). Our integrated scRNA-seq dataset also confirmed that EHF expression of epithelial cells was elevated in tumors, compared with normal and adjacent samples (Fig. 4k). Consistently, in vivo results from IHC staining for EHF in mouse lungs suggested that shJMJD6 A549 tumors had lower tumor expression of EHF (Fig. 4l). Taken together, JMJD6 could promote EHF expression by inducing H4R3me2s demethylation at EHF promoter.

### EHF knockdown inhibits post-RT metastasis of NSCLC cells via TGF-β/ SMAD and AKT/ERK signaling pathways

**Fig. 5:**
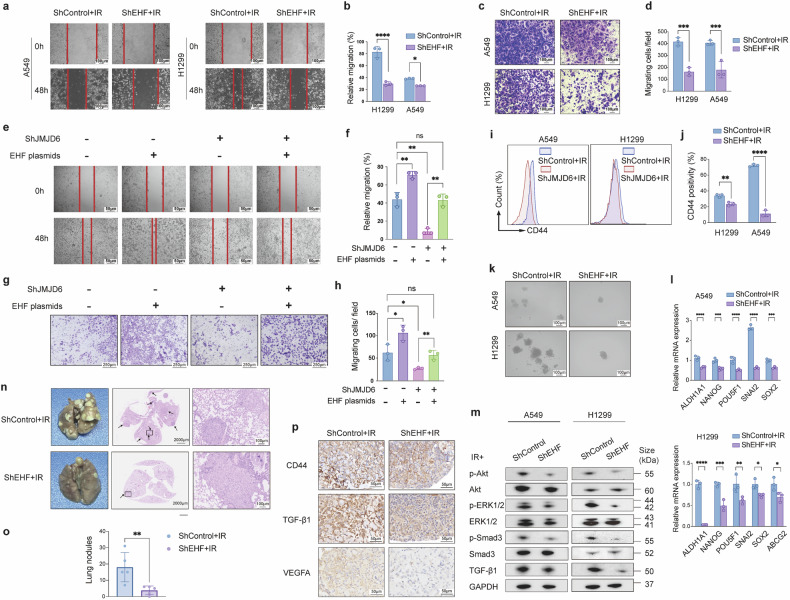
EHF knockdown inhibits post-RT metastasis of NSCLC via TGF-β/Smad and AKT/ERK signaling. **a** Representative images of wound healing assays on A549 and H1299 cells with EHF knockdown, and images were taken at 0 and 48 h after wounds (scale bar = 100 μm). **b** The quantification analyses of relative wound migration. Data shown as mean ± SD, *p < 0.05, ****p < 0.0001. **c** Representative images of transwell assays showing cell migration of A549 and H1299 cells with EHF knockdown (scale bar = 100 μm). **d** Quantification analyses of migrated cells/field. Data shown as mean ± SD, ***p < 0.001. Representative images and quantification of wound-healing (**e**, **f**) and transwell (**g**, **h**) assays. ShJMJD6-H1299 cells were transfected with empty vector or EHF-overexpression plasmids. Data shown as mean ± SD, *p < 0.05, **p < 0.01, ns, not significant; scale bar = 50 μm (wound-healing), and scale bar = 250 µm (transwell assay). **i** Flow cytometry assays detecting CD44 positivity of shControl or shEHF A549 and H1299 cells after IR. **j** Quantification analyses of CD44+ subpopulations. Data shown as mean ± SD, **p < 0.01, ****p < 0.0001. **k** Sphere formation assay of A549 and H1299 cells showing reduction of post-RT sphere-forming capacity by EHF knockdown (scale bar = 100 μm). **l** The qPCR analyses of the expression level of stemness-related genes of shControl or shEHF NSCLC cells. Data shown as mean ± SEM, *p < 0.05,**p < 0.01, ***p < 0.001, ****p < 0.0001. **m** EHF knockdown reduced TGF-β/Smad3 expression and AKT/ERK phosphorylation of NSCLC cells, as assessed by western blot analyses. **n**, **o** IR-treated shControl or shEHF A549 cells were injected through the lateral tail vein of BALB/C nude mice. Mice were sacrificed on the 12th week after injection, and lung metastases were evaluated by gross morphology of the lungs and the number of metastatic nodules on the lung surface (*n* = 5 mice per group). **n** Representative images of H&E staining of mouse lungs with shControl or shEHF A549 tumors, scale bar = 2000 μm (left panel) and 100 μm (right panel). **o** The quantification analyses of metastatic lung nodules. Data shown as mean ± SD, **p < 0.01. **p** Representative IHC staining of CD44, TGF-β1 and VEGFA1 in mouse lungs with shControl or shEHF A549 tumors (scale bar = 50 μm)

To determine the effect of EHF on post-RT metastasis, wound healing, and transwell migration assays were conducted. The knockdown of EHF significantly reduced the wound gap closure rates of both A549 and H1299 cells (Fig. 5a, b). NSCLC cells transfected with shControl or shEHF were placed in transwell chambers after IR. The knockdown of EHF led to decreased migration rates of both A549 and H1299 cells across the chamber (Fig. 5c, d). To confirm that EHF is the downstream effector of JMJD6 in radiation-induced lung-cancer metastasis, we transfected shJMJD6 A549 cells with an EHF-overexpression plasmid and monitored cell migration. In accordance with earlier results, JMJD6 knockdown alone markedly reduced wound healing (Fig. 5e, f) and decreased transwell-migrated cells (Fig. 5g, h). Notably, re-expression of EHF fully reversed this inhibition, restoring migration to levels comparable to control cells. These rescue data demonstrated that JMJD6 promoted lung cancer metastasis predominantly through transcriptional up-regulation of EHF. The proportion of CD44+ subpopulations of shEHF-A549 and shEHF-H1299 cells was also decreased compared with shControl cells (Fig. 5i, j). Sphere formation assays were performed, which suggested that EHF deficiency impaired the sphere formation capacity of NSCLC cells (Fig. 5k). Results from q-PCR assays suggested the downregulation of stemness-related genes of NSCLC cells, including ALDH1A1, POU5F1, SNAI2, and SOX2 by EHF knockdown (Fig. 5l). These results suggested that EHF was positively associated with the metastasis and stemness properties of NSCLC cells.

Activation of the TGF-β/SMAD axis has long been recognized to facilitate the metastatic capacity of cancer cells,^31–33^ we next determined whether the inhibition of tumor metastasis by EHF knockdown was mediated by the TGF-β/SMAD signaling. As shown in Fig. 5m, shEHF-A549 cells and shEHF-H1299 cells revealed reduced levels of Smad3 phosphorylation and TGF-βexpression. Previous studies suggested that EHF deficiency could result in reduced AKT and ERK phosphorylation.^34^ We evaluated the activation of AKT and ERK with Western blot assays, which demonstrated decreased levels of phosphorylated-AKT and -ERK in EHF-knockdown cells, while total protein expression of AKT or ERK was not altered (Fig. 5m). These results confirmed that EHF may regulate the metastasis of NSCLC cells via the TGF-β/ SMAD and AKT/ERK cascades.

### EHF knockdown inhibits post-RT metastasis of NSCLC in vivo

To investigate the role of EHF in the metastatic capacity of NSCLC in vivo, IR-treated shControl- or shEHF-A549 cells were injected through the lateral tail vein of BALB/C nude mice. The numbers of lung metastatic nodules formed by shEHF-A549 cells were significantly reduced compared to those formed by control cells (*P* < 0.01) (Fig. 5n, o, Supplementary Fig. 8). The H&E staining of mouse lung tissues demonstrated that EHF knockdown could suppress the formation of A549 lung metastatic nodules by A549 cells receiving IR treatment (Fig. 5n). Immunohistochemistry staining for CD44, TGF-β, and VEGFA1 was performed in lung tissues of tumor-bearing mice, which demonstrated decreased positivity of CD44, TGF-β1 and VEGFA1 compared with control tumors (Fig. 5p). Thus, EHF is positively associated with cancer stemness, angiogenesis and metastatic abilities of NSCLC tumors.

## Discussion

Radioresistance is a significant clinical challenge in lung cancer that often leads to fatal outcomes, thereby limiting the long-term tumor control after radiotherapy (RT). We herein report for the first time that radioresistance of NSCLC is at least in part driven by JMJD6. Elevated levels of JMJD6 in tumors are linked to unfavorable outcomes and accelerated disease progression across various cancer types, including melanoma,^17^ prostate^10^ and breast cancer.^19^ Through the analysis of a matched human NSCLC tissue array, we observed upregulation of JMJD6 in radioresistant NSCLC cells and patient tumors, which is indicative of a worse prognosis and an elevated risk of metastasis. This finding was further corroborated by our in vivo models, where xenografts derived from JMJD6-deficient cells demonstrated significantly accelerated metastatic spread to distant organs following radiation. In the bulk RNA-seq dataset, elevated JMJD6 expression is associated with worse survival of LUAD patients, whereas this correlation was not significant in LUSC. This histological subtype-specific discrepancy is intriguing and may reflect the distinct cellular origins and molecular pathways that drive LUAD versus LUSC.^35^ The glandular origin of LUAD might be more susceptible to JMJD6-mediated epigenetic reprogramming that promotes stemness and invasion, whereas the squamous lineage of LUSC may rely on alternative resistance mechanisms. We demonstrate that JMJD6 knockdown suppresses the post-RT metastatic potential of NSCLC by modulating the demethylase-dependent transcription of the ETS transcription factor EHF and the expression of a profile of genes linked to cell stemness. These results highlight JMJD6 as a promising therapeutic target to attenuate post-RT metastasis and enhance the long-term effectiveness of RT in lung cancer.

It is well established that radiotherapy is an effective treatment strategy for cancer. It is also reported in the present study that radiotherapy has an anti-tumor effect on bulk populations. While IR suppresses metastatic burden, it concurrently selects for specific subclones with augmented metastatic capacity. The initial cytoreduction of RT provides a selective pressure that eliminates therapy-sensitive cells, while creating a permissive niche that favors the expansion of pre-existing or newly induced resistant clones.^36^ A potential mechanism underlying JMJD6-mediated radioresistance in lung cancer involves the intrinsic presence or the post-IR emergence of CSCs. CSCs, defined by robust self-renewal and multi-lineage differentiation, are recognized drivers of treatment resistance and failure.^37^ Radiation induces the transformation of non-stem cancer cells into CSCs, thereby conferring resistance to RT.^9,10^ This process, often termed cellular plasticity, is increasingly recognized as a major adaptive response to cellular stress, and our experimental results identified JMJD6 as a central epigenetic mediator of this transition. The abundance of CSCs during treatment is a critical determinant for local tumor control following fractionated RT and serves as a prognostic marker for patients undergoing RT.^11,12^ In addition, studies also suggested that CSC populations possess metastatic potential.^38^ CSCs often share molecular programs with embryonic stem cells and EMT, enabling the motility, invasiveness, and the ability of cells to seed and regenerate tumors at secondary sites. CSCs can be identified by specific cell surface markers, such as CD44 and CD133 in lung cancer and CD24 in breast cancer.^39,40^ In our study, CSC subpopulations in IR-treated NSCLC cells exhibited higher JMJD6 expression compared to non-CSC populations, and JMJD6 knockdown attenuated the stemness properties of NSCLC cells in response to IR, providing functional evidence for its role in maintaining the self-renewing CSC pool.

JMJD6 was first described as a histone arginine demethylase for methylated H4R3 in 2007.^16^ In contrast to lysine methylation, which has been intensively studied, arginine methylation is poorly defined for its role in chromatin structure regulation and gene transcription. The histone dimethyl symmetric H4R3 (H4R3me2s) is associated with transcriptional repression,^41,42^, while histone dimethyl asymmetric H4R3 (H4R3me2a) is linked to transcriptional activation.^43^ JMJD6 is able to remove methyl groups of H4R3me2s, thereby activating the transcription of target genes. Our results indicate a co-upregulation of JMJD6 and EHF, suggesting that JMJD6 may demethylate H4R3me2s at the EHF promoter, enhancing its transcription. ChIP assays in our study confirmed a significant enrichment of JMJD6 at the EHF promoter region, concomitant with a decrease in the repressive H4R3me2s mark, providing direct mechanistic insight into this regulatory relationship. EHF is a member of the epithelial-specific ETS (ESE) transcription factors,^44^, and has been identified as a key driving factor for the progression of colorectal cancers and the maintenance of intestinal stem cells.^45^ In lung cancer, EHF overexpression is associated with poor patient prognosis.^34^ Our study suggested that the increased EHF expression in NSCLC cells could, in part, be attributed to JMJD6 upregulation following IR treatment. This establishes a novel IR-JMJD6-EHF signaling axis in EHF, once transcriptionally activated by JMJD6, acts as a pioneer factor that opens up chromatin and facilitates the expression of a broader network of genes involved in cancer cell stemness and survival.

The TGF-β/SMAD cascade is widely recognized for its function in enhancing the metastatic capacity of tumors. Our study reveals that EHF inhibits the post-RT metastatic capacity of NSCLC cells, particularly through the suppression of TGF-β/SMAD and AKT/ERK cascades. This was evidenced by a significant downregulation of phosphorylated SMAD2/3 and AKT upon EHF overexpression. The convergence of JMJD6/EHF on TGF-β/SMAD and AKT/ERK cascades explains the potent effect on tumor metastasis, as these pathways coordinately regulate EMT, cell motility, and survival in the circulation.^46–48^ While our data establish JMJD6 as a critical IR-induced effector, the precise mechanism driving its upregulation warrants further investigation. Potential upstream regulators could include transcription factors activated by the DNA damage response, such as p53 and NF-κB,^49^ or post-transcriptional mechanisms such as mRNA stabilization.^50,51^ Furthermore, the tumor microenvironment, particularly radiation-induced hypoxia or stromal cell interactions, might also contribute to JMJD6 induction, sustaining the resistant phenotype. Elucidating these upstream events will complete the mechanistic landscape from DNA damage to JMJD6-mediated epigenetic reprogramming.

In summary, our findings indicate that JMJD6 contributes to NSCLC radioresistance through the epigenetic modulation of EHF and downstream pluripotency factors. We identified an upregulation of JMJD6 in NSCLC tumors compared to adjacent normal tissues, which correlates with a worse prognosis and higher metastasis risk for NSCLC patients. Given the recent development of several JMJD6 inhibitors, our findings establish JMJD6 inhibition as an effective means to reduce post-RT metastasis and CSC enrichment induced by RT. Future clinical studies are warranted to assess the safety and efficacy of combining pharmacological JMJD6 inhibitors with radiotherapy in the treatment of NSCLC.

## Materials and methods

### Tissue samples

Tumor specimens and clinical data of 188 NSCLC patients were collected from Shanghai Outdo Biotech (National Engineering Centre for Biochip at Shanghai). Among the 188 NSCLC patients enrolled for survival analyses (90 lung squamous cell carcinoma, LUSC, and 98 lung adenocarcinoma, LUAD), 172 patients were enrolled with paired tumor tissues and adjacent normal tissues for the evaluation of JMJD6 expression. Informed consent was obtained from patient,s and the study was approved by the Ethics Committee of the National Human Genetic Resources Sharing Service Platform (permit number: 2005DKA21300). The median survival time was defined as the time duration between initial diagnosis and the final follow-up. Tissue specimens were stained with anti-JMJD6 antibody (Abcam, ab64575) and scored based on the staining intensity and nuclear staining positivity in the field. Tissues were scored as 0 (negative), 1 (1–25%), 2 (26% - 50%), 3 (51–75%), or 4 (76% - 100%), whereas the staining intensity was scored as 0 (negative), 1 (weak), 2 (medium), or 3 (strong). Expression scores were calculated as positivity rates × intensity. Staining scores were independently recorded and represent a consensus between two board-certified pathologists.

### Single-cell RNA-seq data integration

The publicly available scRNA-seq datasets were retrieved from us. FASTQ reads generated with 10x Genomics chemistry were mapped to the GRCh38 reference genome and quantified with Cell Ranger. The resulting count matrices were imported into R by means of Seurat::Read10X (v4.0.4) and base::read.table. Potential multiplet captures were discarded with DoubletFinder. Quality-control filtering removed cells that met any of the following criteria: <200 or >6000 detected genes, or >30% mitochondrial mapping content. Gene counts were log-normalized to equalize library size across cells. Down-stream analysis focused on the 2000 most variable genes selected with FindVariableFeatures. Principal component analysis (PCA) was used to compress the data into 30 major principal components. Residual batch effects were removed with RunFastMNN (SeuratWrappers package Version 0.3.0). Clustering was carried out via FindNeighbors (on the first 30 PCs) followed by FindClusters, and results were visualized with UMAP. The identical procedure was applied for subclustering analysis, including normalization, variable expressed feature, and dimension reduction batch correction. Cluster-specific marker genes were identified with FindAllMarkers using the default Wilcoxon rank sum test with Bonferroni correction.

### Survival analysis

The mRNA expression quantification and corresponding clinical metadata for TCGA cohorts were retrieved from the GDC Data Portal (https://portal.gdc.cancer.gov/). Survival analyses and associated visualizations were performed with the survival (v2.42-3) and survminer (v0.4.9) R packages. Tumor tissues and normal tissues were also retrieved to compare the expression levels of genes.

### Cell lines and cultures

Human NSCLC cell lines, including A549 and H129,9 were purchased from the American Type Culture Collection (ATCC; Rockville, MD, USA). Cells were grown in DMEM (Invitrogen, Carlsbad, CA, USA) medium containing 10% fetal bovine serum (FBS; Gibco, Grand Island, NY, USA), 100 μg/ml streptomycin, and 100 U/ml penicillin. Cell cultures were kept in humidified air at 37 °C with 5% CO_2_.

### Lentiviral transduction and EHF-overexpression transfection

JMJD6- or EHF-knockdown subclones of NSCLC cell lines were established using lentiviral vectors encoding small hairpin RNA (shRNA) targeting JMJD6 or EHF (GeneCopoeia). NSCLC cells were transfected with lentiviral vectors encoding non-targeting control shRNA (shControl), shRNA targeting JMJD6 (shJMJD6), or shRNA targeting EHF (shEHF). Briefly, when NSCLC cells reached 70–90% confluence in the 35 mm well, add lentivirus suspension and incubate at 37 °C. After 16 h, cells were replenished with fresh medium containing 4 μg/mL puromycin (MilliporeSigma) and maintained in puromycin-containing medium for selection of stably transfected clones. For EHF-overexpression transfection, the human EHF open reading frame (NM_001206616) was cloned into pcDNA3.1-3×Flag-C and delivered into H1299 cells with Lipofectamine 3000 (Thermo Fisher). After 48 h of incubation, stable expressors were selected with Geneticin (Thermo Fisher) for 2 weeks to yield the EHF-overexpressing line.

### Flow cytometry

Flow cytometry data were acquired using flow cytometry (ACEA NovoCyte) and analyzed with NovoExpress software. Expression of cancer stem cell markers was evaluated by flow cytometry. Cell debris was first gated out based on cell size, and dead cells were excluded using the Near-IR dead cell stain kit (Invitrogen). Cells were labeled with anti-human CD44 Antibody (BioLegend) and anti-human CD133 Antibody (BioLegend). For the detection of JMJD6 expression, NSCLC cells were first stained with surface markers using the Near-IR dead cell stain kit (Invitrogen) and anti-JMJD6 antibody (Santa Cruz, sc-28348) at 4 °C for 30 min. The surface JMJD6-labeled cells were then fixed and permeabilized by Cytofix/Cytoperm Fixation/Permeabilization Kit (BD Pharmingen, 554715), and stained for intracellular JMJD6 at 4 °C overnight.

### Wound healing and cell migration assays

NSCLC cells were trypsinized and seeded into the six-well plates with 5 × 10^5^ cells per well, and cultured overnight at 37 °C. After cells reach 100% confluence, use a 100 μL pipette tip to draw an artificial wound on the cell monolayer, the images of wound healing were captured at 0 and 48 h. For cell migration assays, trypsinized cells were resuspended in serum-free medium at a cell concentration of 5 × 10^4^ cells/ml. Add 150 μL of the cell suspension added into the upper surface of the wells containing 8-mm pores, and add complete medium containing 10% serum to the wells of the 24-well plates. After 48 h of incubation, cells that migrated through the membranes were fixed with 4% paraformaldehyde at room temperature for 10 min, wash 3 times with PBS, stained with crystal violet for 10 min, and wash 3 times with PBS. Cells that migrated through the membranes were counted under microscopes following air drying at room temperature. Owing to the semi-adherent nature of the murine LL/2 and CMT-64 lung cancer lines, cells adhered to the upper wells could not be reliably counted. Thus, 5 × 10⁴ cells/ml serum-free suspensions (150 µl) were plated in 8-µm-pore inserts and allowed to migrate for 48 h toward 10% serum medium in 24-well plates, and only the cells that had completely traversed and settled in the lower well were counted under microscopes.

### Colony formation assays

JMJD6- or EHF- knockdown subclones of NSCLC cells following 5 Gy ionizing radiation were seeded into 6-well plates for 14 days. Wells were gently rinsed 3 times with PBS, then fixed and stained for 10 min with 0.5% crystal violet. After air-drying at room temperature, colonies were imaged under a light microscope and counted for clonogenic survival analysis.

### Cell viability assays

Cell viability of A549 and H1299 cells following 5 Gy ionizing radiation was assessed with Counting Kit-8 (CCK-8, MCE). In brief, 100 μL of JMJD6- or EHF-knockdown cells were seeded in 96-well plates (1000–4000 cells/well) 24 h after radiation. At the indicated incubation time (24, 48, and 72 h), 10 μL of CCK-8 reagent and 90 μL fresh medium were added to each well and incubated for 1–4 h at 37 °C. Absorbance at 450 nm was measured with microplate readers.

### Sphere-formation assay

Cells transfected with shRNA-JMJD6 or shRNA-control received 5 Gy ionizing radiation. Cells were then collected by trypsinization and seeded in ultra-low cluster 35 mm dishes (Corning, Corning, NY, USA). The DEME/F12 (1:1) medium (Gibco™) supplemented with 2% B27 (Gibco™), 20 µg/ml EGF (Peprotech), and 20 µg/ml of bFGF (Peprotech) was used as culture media. After 10 days, the size of spheroid colonies was measured under optical microscopes, and spheres with a diameter exceeding 50 μm were counted.

### Real-time quantitative PCR (RT-qPCR)

Total RNA was isolated using the Cell Total RNA Isolation Kit (FOREGENE, RE-03113) according to the manufacturer’s protocol. Prime Script RT Kit (Takara, RR047A) was used for reverse transcription to cDNA. Quantitative PCR analysis was performed on the CFX Connect Real-Time PCR system (Bio-Rad, USA) using SsoFast EvaGreen Supermix (Bio-Rad, USA, 1725201). The sequences of the primer sets are provided in Supplementary Table 1.

### Western blot

For total cellular protein extraction, the cells were lysed in RIPA buffer (Beyotime), with protease inhibitor cocktail (MedChemExpress, MCE) and phosphatase inhibitor cocktail (MCE). Cells were ground for 30–60 s (Homogenizer, Servicebio KZ-II), and the supernatant was collected after centrifugation. The quantitative assays of proteins were performed with the PieceTM Rapid Gold BCA Protein Assay Kit (Thermo Fisher, USA, 23225). The mixture of cell lysates and Loading buffer (Beyotime) was denatured in a boiling bath. Equal amounts of protein and molecular weight marker were loaded into the proper sodium dodecyl sulfate-polyacrylamide gel electrophoresis (SDS-PAGE) gels of appropriate concentration, and transferred onto polyvinylidene difluoride (PVDF) membranes. After blocking with 5% skim milk, membranes were incubated with corresponding primary antibodies overnight at 4 °C. Membranes were washed 3 times with 0.1% Tween-20 (TBS-T) and incubated with secondary antibodies for 1 h at room temperature. Band images were acquired with chemiluminescence (Bio-Rad Laboratories). For the detection of protein expression in NSCLC cells following radiation treatment. ShControl or shJMJD6 transfected NSCLC cells received 5 Gy ionizing radiation, and incubated for 48 h. Cells were collected and washed with cold PBS buffer after the indicated treatments. Western blot assays were performed using the anti-JMJD6 antibody (Abcam, ab64575), anti-EHF antibody (Abcam, ab272671), anti-histone H4R3me2s (symmetric) antibody (Active Motif, 61988), anti-GAPDH (Santa Cruz, sc-32233), TGF beta 1 (Abcam, ab215715), phospho-Smad3(S423/425) (Epitomics 1880-1), SMAD3 (1735–1, Epitomics), ERK1/2 (ET1601-29), p44/42 (Erk1/2) (CST, 4695S), Akt (CST, 4691S), and phospho-AKT1 (Ser473) (ThermoFisher, 700392).

### Animal experiments

Animal studies were conducted under guidelines for animal welfare by the Institutional Animal Care and Use Committee of Sichuan University. Human NSCLC cell lines A549 cells (1 × 10^6^ cells/100 µL) were injected intravenously (i.v.). into the tail vein of 6-week-old female BALB/c nude mice (*n* = 5 per group). Mice were divided into four groups according to the cells injected : (1) shControl transfected A549 cells; (2) shJMJD6 transfected A549 cells; (3) shControl transfected A549 cells receiving 5 Gy ionizing radiation; (4) shJMJD6 transfected A549 cells receiving 5 Gy ionizing radiation. Mice were sacrificed 12 weeks after inoculation, and lungs were collected. For better identification of nodules, lungs were fixed in Bouin’s solution for 30 minutes at room temperature, and then fixed with 4% paraformaldehyde. The number of tumor nodules in the lungs was counted under a dissecting microscope. For the animal model using murine cells, mouse Lewis lung carcinoma (LL/2) cells were collected at a concentration of 5×10⁵ cells per 100 µL. Six-week-old female C57BL/6 mice were assigned randomly to two groups (n = 5 per group): (1) shControl-transfected LL/2 cells; (2) shJMJD6-transfected LL/2 cells. Mice were checked daily for signs of morbidity and sacrificed 3 weeks post-inoculation. Lungs were excised and fixed in Bouin’s solution for 15 min at room temperature, followed by overnight fixation in 4% paraformaldehyde. The number of surface tumor nodules on the lungs was counted.

### Hematoxylin-eosin (H&E) and immunohistochemistry (IHC) staining

H&E staining was performed with H&E Staining Kit (Solarbio, G1120) according to the manufacturer’s instructions. Staining of metastatic lung nodules was observed under the microscope. In brief, tissue sections were dewaxed in xylene twice for 5–10 min, and rehydrated with serial ethanol (100%, 95%, 85%, 75%) followed by a distilled water bath for 2 min. Stain the sections with hematoxylin solution for 20 minutes, and wash with distilled water. After differentiation and washing, place sections in the eosin staining solution for 2 minutes and dehydrate sections quickly. For IHC staining, tissue sections were blocked with endogenous peroxide in the dark after dewaxing and rehydration. After blocking slides with goat serum, tissues were incubated with primary antibodies. The slides were then incubated with the HRP-conjugated secondary antibody and streptavidin-biotin complex. HRP was detected with diaminobenzidine peroxide. IHC assays were performed using the anti-EHF antibody (Abcam, ab272671), CD44 (Abcam, ab189524), TGF-β1 (Proteintech, 21898-1-ap), and VEGFA1(Proteintech, 19003-1-AP).

### RNA-sequencing (RNA-seq) analysis

RNA-seq analyses were performed comparing the differential expression of genes in shControl-transfected A549 cells and shJMJD6-transfected A549 cells treated with 5 Gy ionizing radiation. After 24 h, shControl- or shJMJD6-transfected A549 cells that received 5 Gy ionizing radiation were lysed in TRIzol (Invitrogen, 15596026), with three biological duplicates for each condition. The integrity and concentration of RNA extracts were determined by Agilent 2100 Bioanalyzer and RNA Nano 6000 Assay Kit (Agilent Technologies), and RNA integrity numbers ranged between 8.3 and 9.7. To prepare an RNA-seq library, total RNA was purified by oligo (dT) beads and fragmented, followed by synthesis of first and second strands, 3′ ends adenylation, and adapter ligation. Afterwards, samples were amplified by PCR, subsequently by gel extraction. Libraries were analyzed on Illumina HiSeq 2500 (Illumina) following a PE150 sequencing strategy.

### CUT&Tag assay and data analysis

The CUT&Tag assay was performed using a Novaseq 6000 sequencer (Illumina) with the PE150 model. The Hyperactive universal CUT&Tag assay kit for Illumina (Vazyme, TD903) was used to collect cells. In brief, 50,000 A549 cells were collected and incubated with Concanavalin A-coated magnetic beads. Next, we incubated cells with ChIP-grade primary antibodies: anti-JMJD6 antibody (Abcam, ab272671) or anti-histone H4R3me2s (symmetric) antibody (Active Motif 61988) for 2 h, followed by secondary antibody incubation for 1 h at room temperature. After fragmentation with hyperactive pG-Tn5 transposase, the DNA fragments were extracted, and sequencing libraries were generated by PCR amplification using indexed P5 and P7 primers. Raw sequencing data were first filtered by FASTP (version 0.23.1) to exclude low-quality reads. Clean reads were mapped to the reference genome of the human (hg19). To identify the potential binding region by JMJD6 on the EHF genome, the purified DNA was subjected to quantitative real-time PCR analyses with the indicated primers. Linear equations based on the serial dilutions of the standard EHF plasmids were used to calculate the absolute quantity of DNA, presented as Log [copy number]. The sequence of PCR primers and EHF plasmids is provided in Supplementary Tables 2, 3.

### Statistical analysis

All statistical analyses were performed using GraphPad Prism 7.0 and IBM SPSS Statistics 26.0. Kaplan-Meier survival analyses and log-rank tests were performed to evaluate the correlation between clinicopathological characteristics and patient prognosis. The survival time of patients was presented as median ± SD. The significance of differences between the 2 groups was determined by Student’s *t*-test (parametric) and ANOVA multiple comparison tests. For comparisons of tumor volumes across more than two groups, we used one-way ANOVA followed by Tukey’s multiple-comparison post-hoc test (Shapiro-Wilk p > 0.05) and homogeneity of variance (Levene’s *p* > 0.10). Non-normally distributed metastasis counts were analyzed by the Kruskal-Wallis test with Dunn’s correction. All analyses used two-tailed tests with α = 0.05. Comparison of JMJD6 expression scores between NSCLC tissues and paired non-cancerous tissues was determined using the Wilcoxon matched-pairs signed-rank test. Comparisons of the tumor JMJD6 levels between metastatic and non-metastatic patients were determined using the Mann-Whitney test. Statistically significant *p* values were labeled as: *p < 0.05, **p < 0.01, ***p < 0.001, ****p < 0.0001.

## Supplementary information

SUPPLEMENTAL MATERIAL

## Data Availability

The dataset supporting the conclusions of this article is included within the article and its additional files. The data in this study are available in Genome Sequence Archive in BIG Data Center, Beijing Institute of Genomics, Chinese Academy of Sciences (https://ngdc.cncb.ac.cn/gsub/, access number: PRJCA021707) (10) and Gene Expression Omnibus (GEO) at GSE227136, GSE131907,^52^ GSE134355,^22^ PRJNA668853, and E-MTAB-8530.^25^
