# Supplementary material for: Integrative analysis of non-small cell lung cancer identifies Jumonji domain-containing 6/ETS homologous factor axis as a target to overcome radioresistance
Source: Signal Transduct Target Ther. 2025 Dec 1;10:391. doi: 10.1038/s41392-025-02471-w (PMC12665795; doi:10.1038/s41392-025-02471-w)
Supplement: Supplementary file 1 — SUPPLEMENTAL MATERIAL [file 41392_2025_2471_MOESM1_ESM.docx]

Supplementary Materials for

Integrative analysis of non-small cell lung cancer identifies Jumonji domain-containing 6 /Ets homologous factor axis as a target to overcome radioresistance

Manni Wang 1,2#*, Li Xu 1#, Aqu Alu 1#, Peiheng Li 3#, Jian Liu 1, Siyuan Chen 1, Xuemei He 1, Xuejiao Han 1, Li Yang 1*, Qiang Pu 4*, Xiawei Wei 1*

Correspondence to: Xiawei Wei: xiaweiwei@scu.edu.cn; Qiang Pu: Qiang Pu, puqiang100@163.com; Li Yang: yl.tracy73@gmail.com, and Manni Wang: wangmanni@scu.edu.cn.

**This PDF file includes:**

Figures. S1 to S8

Tables S1 to S3


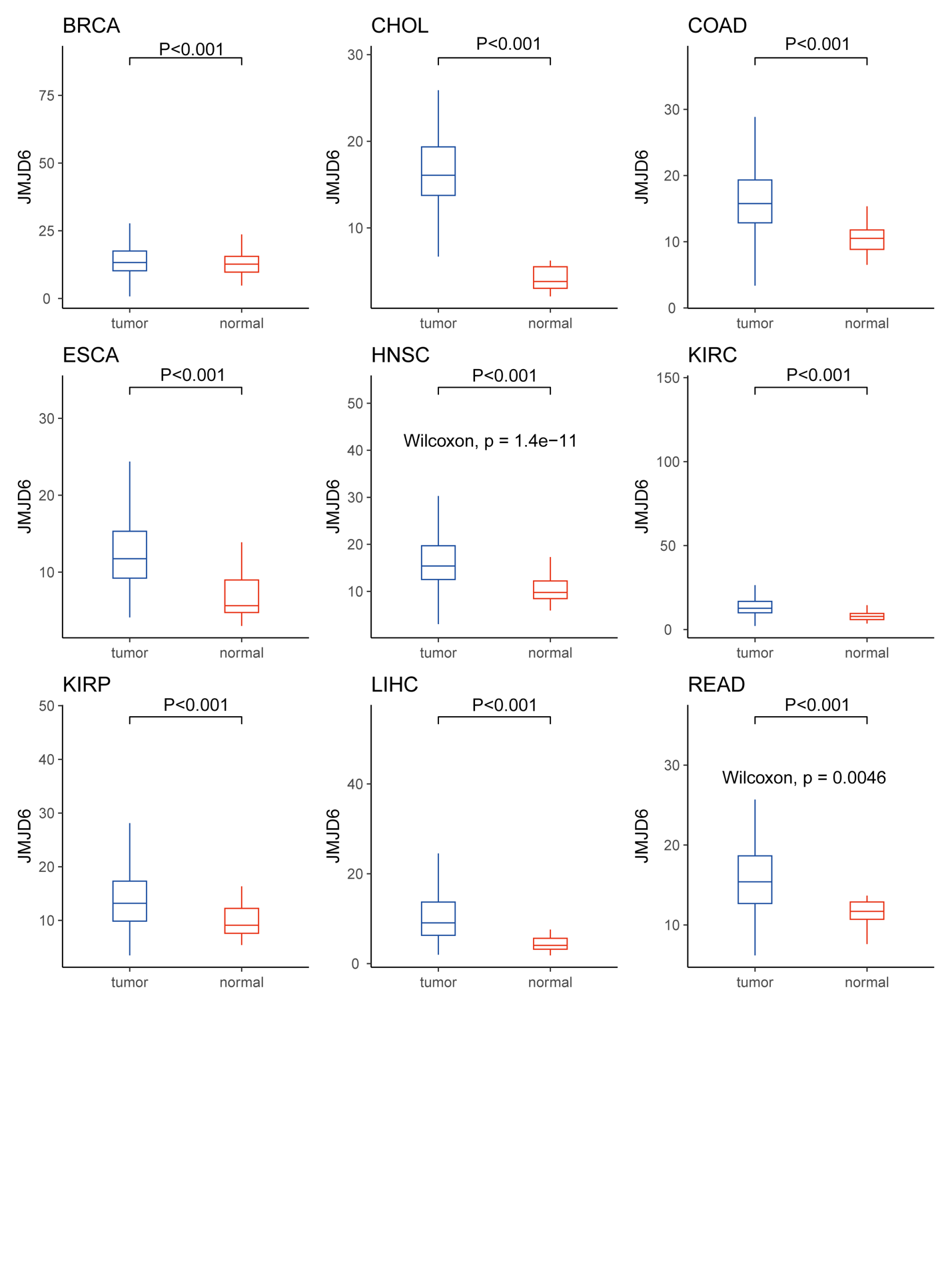
Figure. S1.

The boxplots displaying the expression of JMJD6 in tumor and normal samples from other cancer types using RNA-seq data derived from TCGA. BRCA, Breast Cancer. CHOL, Cholangiocarcinoma. COAD, Colon adenocarcinoma. ESCA, Esophageal carcinoma. HNSC, Head and Neck squamous cell carcinoma. KIRC, Kidney renal clear cell carcinoma. KIRP, Kidney renal papillary cell carcinoma. LIHC, Liver hepatocellular carcinoma. READ, Rectum adenocarcinoma.


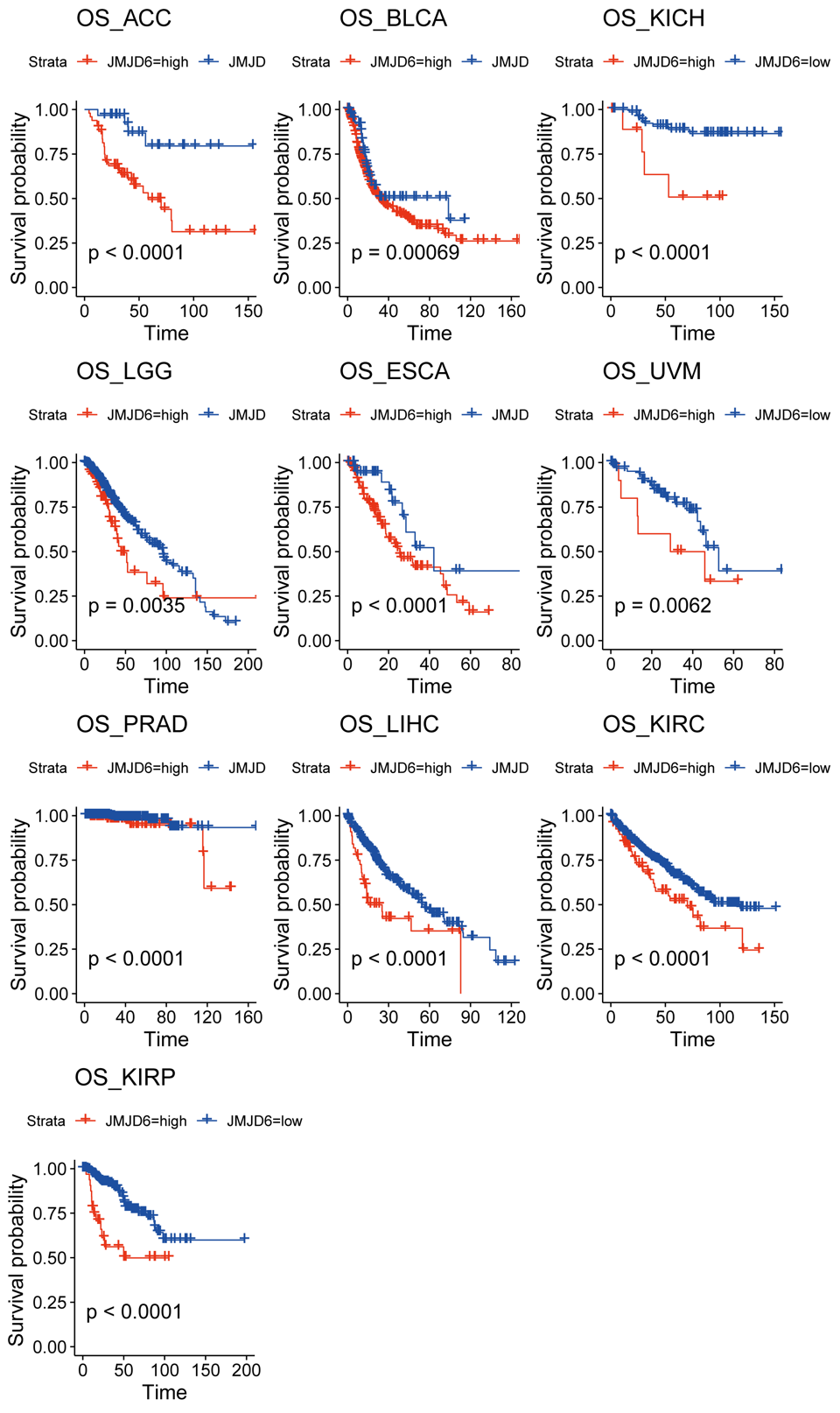


Figure. S2.

The survival curve of the JMJD6 in patients of other cancer types using TCGA dataset. ACC, Adrenocortical carcinoma. BLCA, Bladder Urothelial Carcinoma. KICH, Kidney Chromophobe. LGG, Brain Lower Grade Glioma. ESCA, Esophageal carcinoma. UVM, Uveal Melanoma. PRAD, Prostate adenocarcinoma. LIHC, Liver hepatocellular carcinoma. KIRC, Kidney renal clear cell carcinoma. KIRP, Kidney renal papillary cell carcinoma.


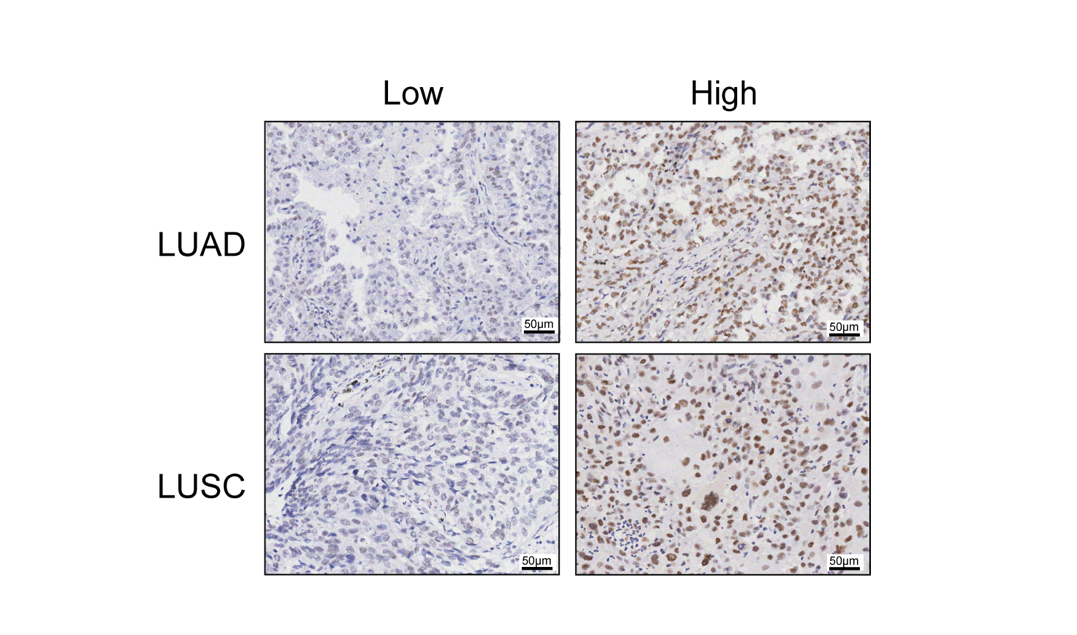


Figure. S3.

The example of high and low expression of tumor tissues (scale bar = 50 μm). LUAD, lung adenocarcinoma. LUSC, lung squamous cell carcinoma.


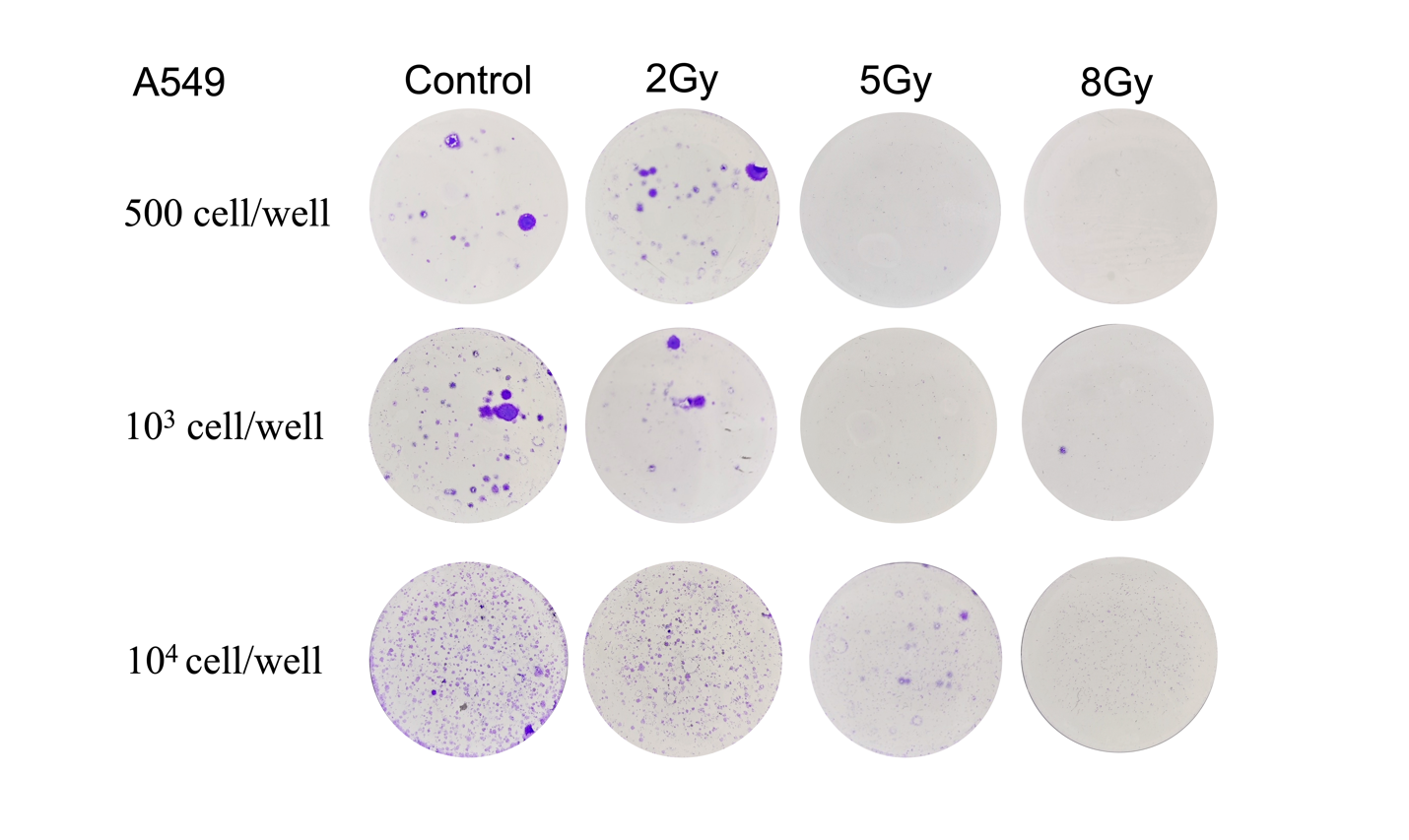


Figure. S4.

Representative images of colony formation of A549 cells receiving RT of 0, 2, 5, and 8 Gy.


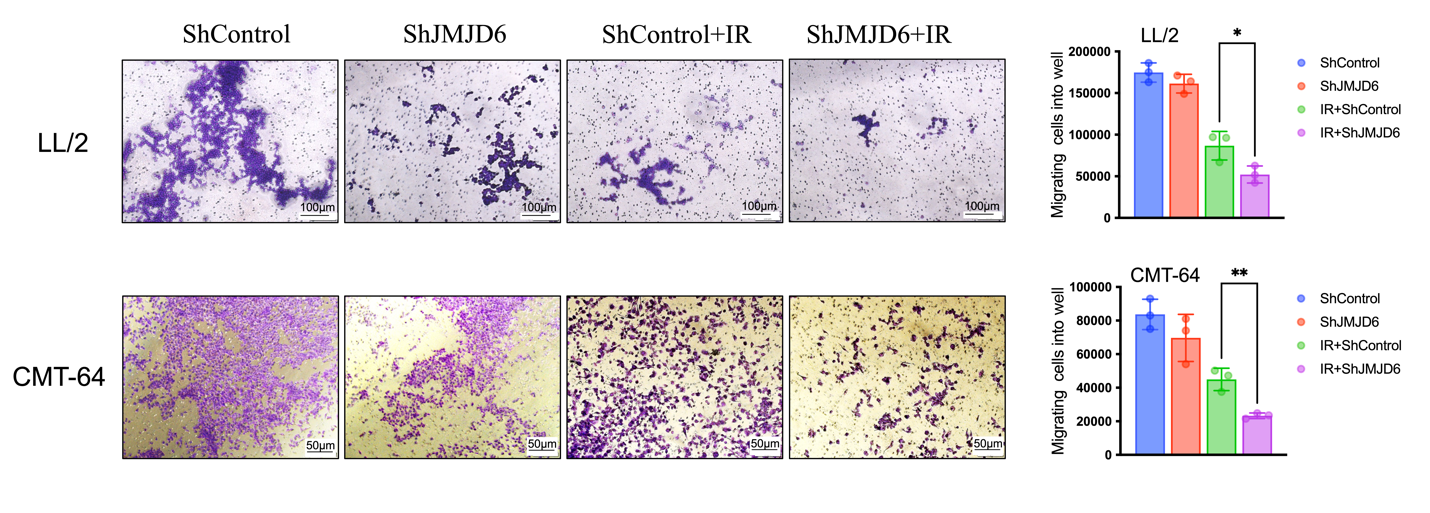


Figure. S5.

JMJD6 knockdown decreased the cell migration of LL/2 and CMT-64 murine lung cancer cells receiving RT.


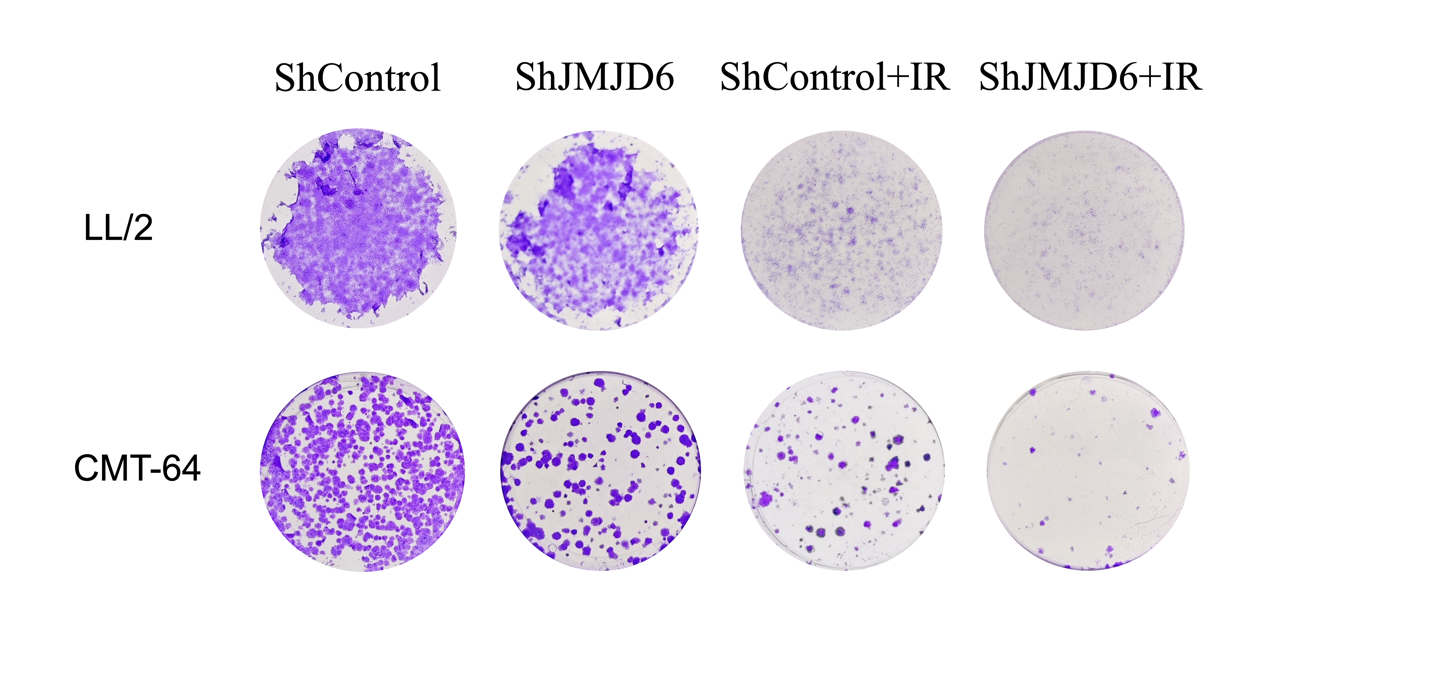


Figure. S6.

JMJD6 knockdown decreased the colony formation of LL/2 and CMT-64 murine lung cancer cells receiving RT.


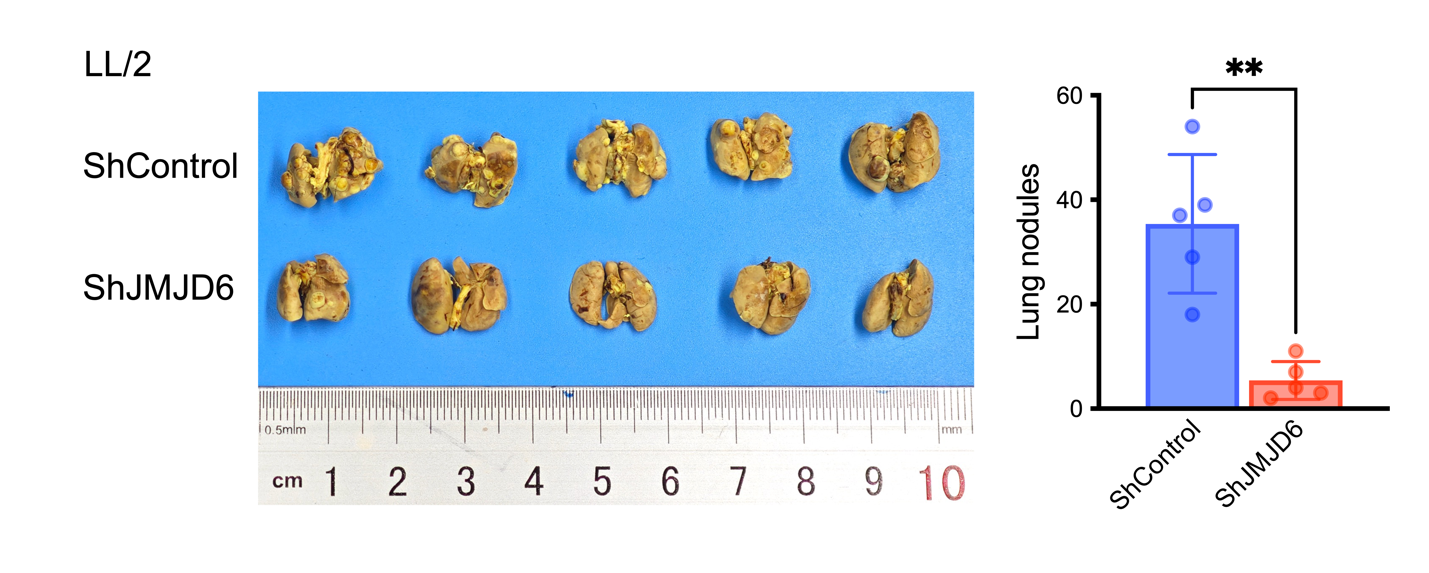


Figure. S7.

Mice inoculated with shJMJD6 LL/2 murine lung cancer cells developed decreased pulmonary metastatic foci than shControl group.


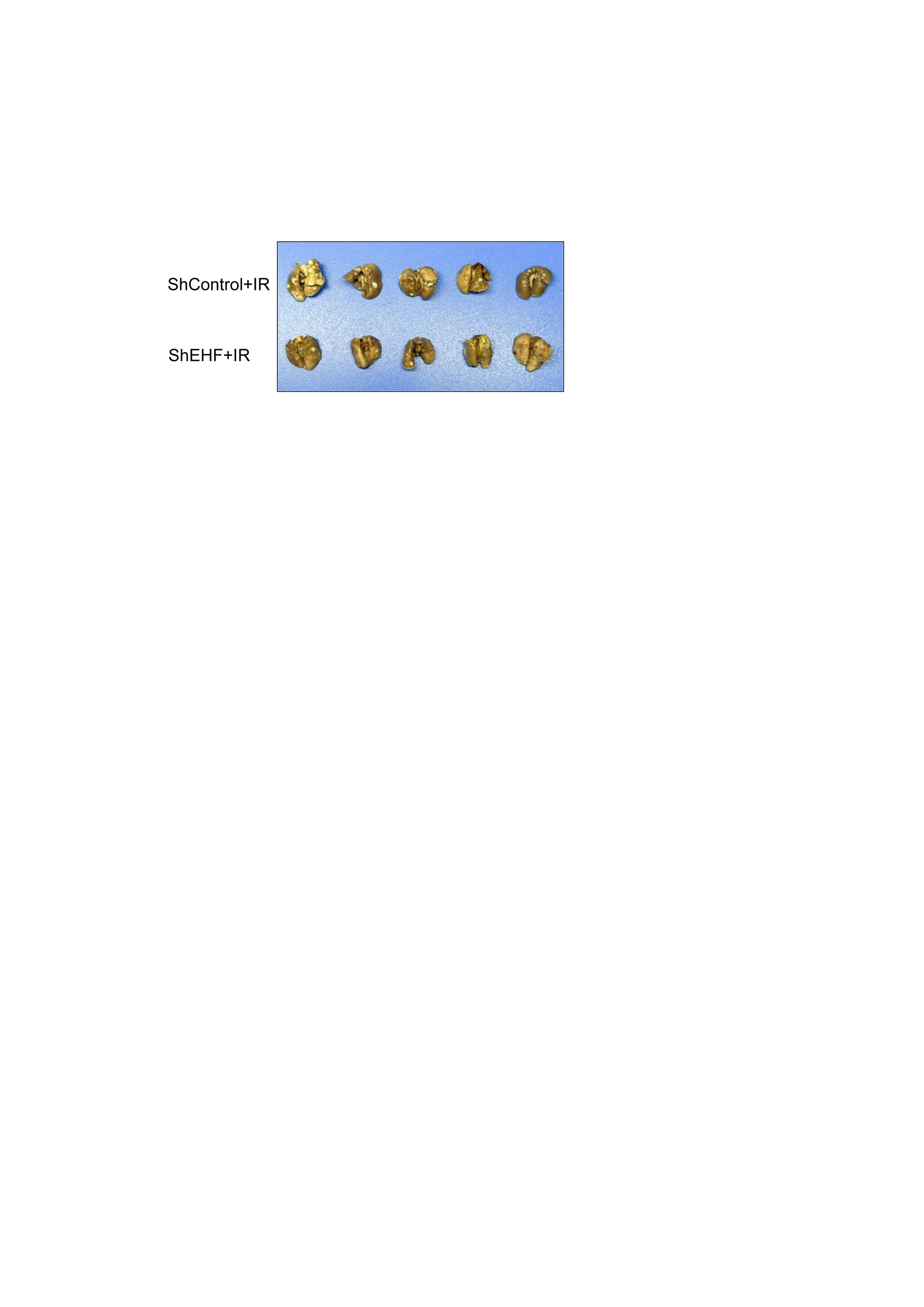


Figure. S8.

Mice inoculated with shEHF A549 cells developed decreased numbers of pulmonary metastatic foci than shControl group.

Table S1.

The sequences of the primer sets used in real-time quantitative PCR assays.

|  | Forward | Reverse |
| --- | --- | --- |
| GAPDH | GTCTCCTCTGACTTCAACAGCG | ACCACCCTGTTGCTGTAGCCAA |
| JMJD6 | CCAACTTCCCTGTGGTATGGCA | TCCTGAAGGTCAACCGAGTCTG |
| ABCG2 | GTTCTCAGCAGCTCTTCGGCTT | TCCTCCAGACACACCACGGATA |
| ALDH1A1 | CGGGAAAAGCAATCTGAAGAGGG | GATGCGGCTATACAACACTGGC |
| SNAI2 | ATCTGCGGCAAGGCGTTTTCCA | GAGCCCTCAGATTTGACCTGTC |
| POU5F1 | CCTGAAGCAGAAGAGGATCACC | AAAGCGGCAGATGGTCGTTTGG |
| NANOG | CTCCAACATCCTGAACCTCAGC | CGTCACACCATTGCTATTCTTCG |
| SOX2 | GCTACAGCATGATGCAGGACCA | TCTGCGAGCTGGTCATGGAGTT |

Table S2.

The sequence of PCR primers used for CUT&Tag real-time quantitative PCR analyses.

|  | Forward | Reverse |
| --- | --- | --- |
| Primer 1 | 5'-CAGCTATAAATTACAGGGCCT-3' | 5'-GGTGGGAGTAGAAGGGCTA-3' |
| Primer 2 | 5'-GGGGCTAATGTGATAATGGG-3' | 5'- AACCCTGTGAACTTGCCAA-3' |
| Primer 3 | 5'-TAGAGGTTGCCTGGGTATTT-3' | 5'- GAGTTAGAGATGAGAAGACC-3' |
| Primer 4 | 5'-CCATCATCTCCTCCAGATTTGG-3' | 5'- CAGAGGCTGTTTCAGGTGAA-3' |

Table S3.

The sequence of standard EHF plasmids.

| EHF1 | CAGCTATAAATTACAGGGCCTATAATTAAAGGTGATTGGGACTGGGTCAGAGAGCCACATCACTTTTGTGGTTGCATTTGAAGTTCACTATCTCTTGACCACACAACCCTAGCCCTTCTACTCCCACC |
| --- | --- |
| EHF2 | GGGGCTAATGTGATAATGGGAAATAATGAAATTTGTTGTTTTTATCAGTGTGTATATGGGGCGGGGTTTACATTTGCATTTTCACAGGGCCCTTGGCAAGTTCACAGGGTT |
| EHF3 | TAGAGGTTGCCTGGGTATTTGAATCCGTAGATCCTCCCTAATATTCCACCTTCTTCTTGTCCAAACTGTGCTTTTTTATTTCCAGTTTCAGCATTTTGGTCTTCTCATCTCTAACTC |
| EHF4 | CCATCATCTCCTCCAGATTTGGACTTCTACTCACTTTGCTTTTACATTCCCTCTTCCCGATGGTGTCTTTGGTGAGCAGGGTGCTTTTCACCTGAAACAGCCTCTG |
